# Corrigendum to “Mechanisms and Pathophysiological Significance of Insulin Resistance in Offspring With Intrauterine Growth Restriction Mediated by Hepatic GR/miR‐1224 Programming”

**DOI:** 10.1002/advs.76952

**Published:** 2026-08-05

Y. Dai, X. Guo, P. Yu, D. Zhang, H. Kou, and H. Wang, “Mechanisms and Pathophysiological Significance of Insulin Resistance in Offspring With Intrauterine Growth Restriction Mediated by Hepatic GR/miR‐1224 Programming,” *Advanced Science* 12, no. 44 (2025): e10277, https://doi.org/10.1002/advs.202510277.

In the “Acknowledgements” section, one grant number from the National Natural Science Foundation of China was incorrectly listed as “82414020.” The correct grant number should be “82474020.” This correction does not affect the results, discussion, or conclusions of the article.

The authors apologize for this error.

